# Governing health professional mobility in the European Union

**DOI:** 10.1186/1472-6963-14-S2-O33

**Published:** 2014-07-07

**Authors:** Matthias Wismar

**Affiliations:** 1 European Observatory on Health Systems and Policies, Brussels, Belgium

## 

Health professional mobility in Europe has become a fast-moving target for policy-makers. It is evolving rapidly in direction and magnitude as a consequence of fundamental change caused by European Union (EU) enlargement and the financial and economic crisis.

Health professional mobility changes the numbers of health professionals in countries and the skill-mix of the workforce, with consequences for health-system performance. Many of the so-called pull and push factors influencing mobility of individuals are in the remit of countries and organizations. In Europe, these pull and push factors are co-determined by EU policies on free mobility, the qualifications directive and many soft-law initiatives.

This presentation is reviewing the current mobility trends in Europe and provides clues on how to strengthen governance for human resources for health at European and country level. A particular reference is made to mobility monitoring, workforce intelligence, workforce policies/ strategies, skills-initiatives and coordination mechanisms across sectors and levels.

